# A Narrative Review on the Role of Artificial Intelligence (AI) in Colorectal Cancer Management

**DOI:** 10.7759/cureus.79570

**Published:** 2025-02-24

**Authors:** Bijily Babu, Jyoti Singh, Juan Felipe Salazar González, Sadaf Zalmai, Adnan Ahmed, Harshal D Padekar, Marina R Eichemberger, Abrar I Abdallah, Irshad Ahamed S, Zahra Nazir

**Affiliations:** 1 Clinical Research, Network Cancer Aid and Research Foundation, Cochin, IND; 2 Department of Medicine, American University of Barbados, Bridgetown, BRB; 3 General Medicine, Clínica Renovar, Villavicencio, COL; 4 Emergency Medicine, New York Presbyterian Hospital, New York, USA; 5 Medicine and Surgery, York University, Bradford, CAN; 6 General Surgery, Grant Medical College and Sir Jamshedjee Jeejeebhoy Group of Hospitals, Mumbai, IND; 7 General Surgery, Centro Universitário São Camilo, São Paulo, BRA; 8 Medicine and Surgery, Sulaiman Al Rajhi University, Al Bukayriyah, SAU; 9 General Surgery, Pondicherry Institute of Medical Sciences, Pondicherry, IND; 10 Internal Medicine, Combined Military Hospital, Quetta, PAK

**Keywords:** artifical intelligence, colon capsule endoscopy, colorectal cancer, computed tomographic colonography, microsatellite instability

## Abstract

The role of artificial intelligence (AI) tools and deep learning in medical practice in the management of colorectal cancer has gathered significant attention in recent years. Colorectal cancer, being the third most common type of malignancy, requires an innovative approach to augment early detection and advanced surgical techniques to reduce morbidity and mortality. With its emerging potential, AI improves colorectal cancer management by assisting with accuracy in screening, pathology evaluation, precision, and postoperative care. Evidence suggests that AI minimizes missed cases during colorectal cancer screening, plays a promising role in pathology and imaging diagnoses, and facilitates accurate staging. In surgical management, AI demonstrates comparable or superior outcomes to laparoscopic approaches, with reduced hospital stays and conversion rates. However, these outcomes are influenced by clinical expertise and other dependable factors, including expertise in implementing AI-based software and detecting possible errors. Despite these advancements, limited multicenter studies and randomized trials restrict the comprehensive evaluation of AI's true potential and integration into standard practice. We used Pubmed, Google Scholar, Cochrane Library, and Scopus databases for this review. The final number of articles selected, depending on inclusion and exclusion criteria, is 122. We included papers published in the English language, literature published in the last 10 years, and adult patient populations above 35 years with colorectal cancer. We thoroughly included randomized controlled trials, cohort studies, meta-analyses, systematic reviews, narrative reviews, and case-control studies. The use of AI paves the way for the adoption of more personalized medicine. This review highlights the advantages of AI at various disease stages for colorectal cancer patients and evaluates its potential for cost-effective implementation in clinical practice.

## Introduction and background

Colorectal cancer is the third most common malignancy diagnosed worldwide and is the second leading cause of tumor-related deaths. There were an estimated 1.9 million new cases and 935,000 deaths attributed to colorectal cancer (CRC) worldwide in the year 2020 [1]. The majority of CRC pathogenesis involves genetic alteration sequences of genes, like the adenomatous polyposis coli (APC) gene, which is the most common, followed by Ki-ras2 Kirsten rat sarcoma viral oncogene homolog (KRAS) and tumor protein p53 (TP53), which evolve from benign adenomas to malignant carcinomas and other molecular pathways responsible for driving tumorigenesis. Approximately 60-70% of patients with clinical manifestations of CRC are diagnosed at advanced stages, with liver metastases present in almost 20% of cases [1]. Of that, five-year overall survival rates drop from 80-90% in the case of local disease to a dismal 10-15% in patients with metastatic spread at the time of diagnosis. CRC diagnosis is confirmed by colonoscopy with biopsy, together with imaging techniques such as computed tomography (CT) or magnetic resonance imaging (MRI) for staging. The US Preventive Services Task Force (USPSTF) has recommended preventive screening at 45 years of age for individuals without any risk factors, which is a grade B recommendation, while those with a family history or genetic predisposition should start early [2]. CRC is managed by surgical resection if localized, often combined with chemotherapy or radiation if at an advanced stage. The surgical approach has evolved extensively compared to practice before 2015 and is progressively improving with more artificial intelligence (AI) integration. 

In recent years, artificial intelligence has become an integral part of many fields; its applications in medicine are steadily increasing and have already demonstrated clinical impact in various fields, including dermatology, pathology, and endoscopy [3-5]. Its use, particularly in medical oncology, has dramatically increased and influenced the disease's management in every aspect. AI's role in colorectal cancer is vital in every stage of its management, from detection to surgical management, which significantly depends on AI. Many research studies have shown that the use of AI in colorectal cancer has decreased fatality due to early detection and management [5].

In recent years, AI use has decreased colon cancer rates with early detection and improved screening outcomes. AI has also played a critical role in the surgical management of late or advanced cancer. AI in the medical field is divided into virtual and physical branches. Virtual components include machine learning and deep learning, whereas physical components include medical devices and robots. Machine learning (ML) determines the sequence from already installed data, while deep learning (DL) uses multi-layer neural networks to identify patterns.

The role of AI in colorectal cancer management has become an integral part of screening in preventive medicine. Its uses in colonoscopy, capsule endoscopy, and polyp detections to surgical removal of colon cancer have proven positive overall outcomes of colon cancer. Its role in screening methods by improving the quality of images and ease of analysis has outweighed CT or MRIs. AI has been integral to surgeries and minimally invasive techniques like robotic surgery and laparoscopy.

AI use in colorectal surgery for colon cancer has rapidly expanded in recent years. It has various applications in surgical management, including preoperative imaging analysis for accurate localization of the spread of cancer and predicting the survival rate or success of surgical management. AI has given the golden platform to surgeons by helping with many tasks during surgery, like identifying dissection planes, localizing cancer margins, and generating real-time images during surgery for accurately removing cancer [5,6]. 

With this expansion of AI in every margin of oncology, there is still a lot to learn about its potential in colorectal surgery in the coming future, as it is still at an experimental stage, and more extensive data is needed before its worldwide adoption, especially in colorectal surgery. Nonetheless, AI is enhancing its precision with upcoming new technologies in cancer management and has shown a reduction in mortality. 

## Review

AI's role in colorectal cancer screening

Around the world, CRC is a leading cause of morbidity and mortality. Early detection with a helpful screening test can lower the mortality and morbidity it causes [6]. The goal of regular screening recommendations is to find and remove precancerous polyps before they develop into cancer. Because CRC is a prevalent and preventable cancer, routine screening is recommended. Numerous screening methods are currently in use, including non-invasive techniques such as stool-based tests, namely fecal occult blood test (FOBT), fecal immunochemical test (FIT), and FIT-DNA test; blood-based tests such as the SEPT9 DNA methylation test (Epi proColon®); imaging methods, namely colon capsule endoscopy (CCE), computed tomographic colonography (CTC), double contrast barium enema; and invasive techniques of flexible sigmoidoscopy and colonoscopy. Each of these techniques has slightly different sensitivity and specificity [7]. 

Colonoscopy allows the detection and subsequent removal of precursor lesions (adenomas) of CRC. As the adenoma detection rate (ADR) is considered a significant quality indicator, colonoscopy is accepted as it prevents incidence and mortality from colorectal cancer [8].

Colonoscopy is the gold standard screening test for early cancer and adenomatous polyps. It has some drawbacks, including a high polyp miss rate for tiny (<10 mm) or flat polyps, which are frequently overlooked during inspection [9]. Colonoscopy is highly operator-dependent; therefore, a significant adenoma miss rate (as high as 26%) has been observed. The main reasons are failure to recognize the small, proximal, non-polypoid lesions due to inexperience, fatigue, or distraction of the endoscopists and incomplete exposure of the colorectal mucosa, including patient factors and poor bowel preparation [8,10]. This is why the goal of AI in colonoscopy is to improve the performance of non-expert endoscopists by helping them achieve results comparable to those obtained by experts [8]. 

The automatic polyp identification and classification method is anticipated to enhance the quality of the artificial intelligence assistant system. These AI-powered functions could result in a higher adenoma diagnosis rate and lower polypectomy costs for hyperplastic polyps. Furthermore, artificial intelligence effectively stages, diagnoses, and segments colorectal cancer patients [11]. AI-based computer-aided diagnosis systems, such as deep learning, convolutional neural networks, and machine learning, are used for automated colonoscopy detection and diagnosis of colorectal polyps [12]. 

The endoscopist's ADR fluctuates significantly during colonoscopy. AI can compensate for perceptual errors, reducing performance variability [13]. A multicentre randomized controlled trial (RCT) of asymptomatic individuals by Xu et al. [14] found that AI-assisted colonoscopy raised the overall ADR, advanced ADR, and ADR of both expert and non-expert attending endoscopists. Endoscopists have always strived for the perfect colonoscopy, and the use of AI with deep-learning algorithms is one of the most promising assistance options for detecting and characterizing colorectal polyps during colonoscopy [15].

In order to remove more precancerous polyps, promising techniques for raising adenoma detection rates are provided by AI developments, particularly computer-aided diagnoses. In contrast, these techniques might also make it possible to identify non-cancerous lesions in vivo and leave them in situ, lowering the potential risks associated with unneeded polypectomies [16]. By breaking AI capabilities into three categories - computer-aided detection (CADe), diagnosis (CADx), and quality assessment (CADq) - each application can be systematically evaluated. In addition to identifying the causes of bleeding and disease in the small intestine, CADe investigations have demonstrated promise in diagnosing esophageal, gastric, and colonic neoplasia precisely. Diverse CADq applications guarantee quality and boost procedure efficiency, while more sophisticated CADx applications use optical biopsies to provide additional information to characterize neoplasia and grade inflammatory disease [17]. 

AI computer-aided detection and diagnosis systems consist of data from video recordings of colonoscopies that experts later revise. Then, the lesions' type is histologically confirmed. It is an input-output process; the endoscopy display collects data from the digital frames of the standard endoscopic processor and outputs the coordinates of a signal when a target lesion is recognized in the image [18]. 

A study compared the computer-aided diagnosis (CADx) mode of the CAD EYE system (Fujifilm Corporation, Tokyo, Japan) and the performance of an expert for optical diagnosis of colorectal lesions. A total of 110 lesions (80, 72.7%, dysplastic lesions and 30, 27.3%, non-dysplastic lesions) were evaluated. The artificial intelligence results showed 81.8% accuracy, 76.3% sensitivity, 96.7% specificity, 98.5% positive predictive value (PPV), and 60.4% negative predictive value (NPV). The expert results showed 93.6% accuracy, 92.5% sensitivity, 96.7% specificity, 98.7% PPV, and 82.9% NPV [8].

Colorectal cancer screening outcomes have been encouraging when AI and computer-aided detection are combined with screening techniques. When combined with colonoscopy and other cutting-edge endoscopic techniques, including magnifying narrow-band imaging, endo-cytoscopy, confocal endo-microscopy, laser-induced fluorescence spectroscopy, and magnifying chromo-endoscopy, it can also enhance the identification and analysis of polyps [6]. Using artificial intelligence-based computer-aided polyp detection systems as opposed to high-definition white light (HDWL) colonoscopy alone results in a decrease in the adenoma miss rate (AMR) and sessile serrated lesion (SSL) miss rate as well as an increase in first-pass adenomas per colonoscopy (APC) [19].

Polyp diagnosis and removal during colonoscopy considerably decrease long-term colorectal cancer risk. According to Ahmad et al., CADe increased the polyp detection rate (PDR) but not the adenoma detection rate in high-performing endoscopists who are regularly using Endocuff Vision™ (Olympus Medical Systems Corp., Tokyo, Japan) in the NHS Bowel Cancer Screening Program (BCSP). In a BCSP context where skilled endoscopists carry out procedures, CADe seemed to offer no advantage [20]. AI can potentially reduce operator-dependent variability in colonoscopy quality [21].

Despite being inferior to expert performance, AI provides good accuracy in diagnosing colorectal lesions, which is important in clinical practice. It improves the performance of endoscopists who lack expertise [8]. More than ten randomized controlled trials conducted around the globe assure this. CADe demonstrated superiority in decreasing adenoma miss rates and increasing adenoma detection rates compared with standard colonoscopy [22].

Colonoscopy-missed lesions are a significant cause of post-colonoscopy colorectal cancer, which typically has a poorer prognosis. According to Lui et al., using the deep learning model, AI can lower the miss rate and accurately detect colorectal polyps [10]. Patients undergoing CRC screening or surveillance were the study population enrolled in centers located in Italy, the UK, and the US. They were randomized to undergo two same-day colonoscopies, with and without AI (deep learning computer-aided diagnosis endoscopy), one followed by the other (a total of 230 participants, 116 went to AI first, and 114 were directed to standard colonoscopy first). According to Wallace et al., AI reduced the miss rate of colorectal neoplasia by about two times, indicating that AI can help reduce perceptual errors for smaller and more subtle lesions during routine colonoscopy [23]. An adenoma miss rate was calculated through the number of lesions detected in the second colonoscopy that were not identified at the first colonoscopy (false negative results), and it resulted in 15.5% and 32.4% in the arm with AI and non-AI colonoscopy first, respectively. Going deeper, AMR was lower for AI first for the 5 mm and non-polypoid lesions in the proximal and distal colon [23].

**Figure 1:**
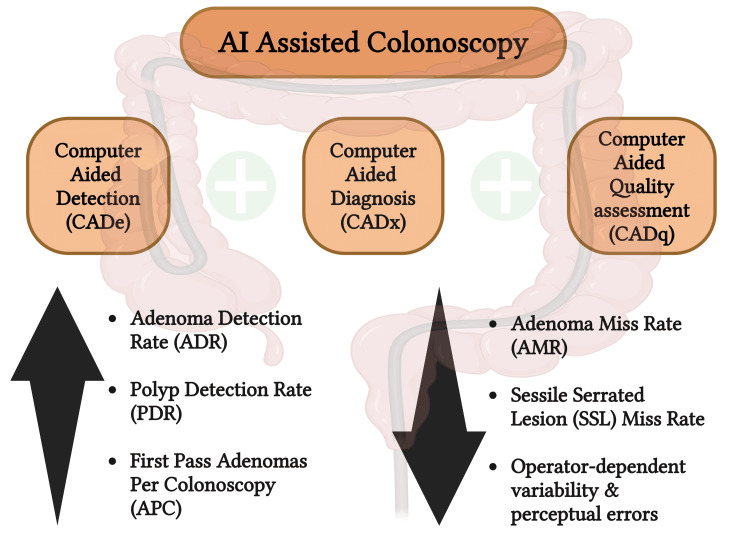
Advantages of AI-assisted colonoscopy The image is created by Bijily Babu using biorender.com.

Mori et al. found that using AI during colonoscopy increased the proportion of US and European patients who required comprehensive colonoscopy surveillance by approximately 35% and 20%, respectively (absolute increases of 2.9% and 1.3%). Even though it may help prevent cancer, this significantly raises patient burden and healthcare costs [24]. The AI-augmented polyp-detection algorithm can detect polyps during a colonoscopy. It assists clinicians in increasing the adenoma identification rate, which could lead to cancer prevention. According to Park et al., the group that used the AI model based on RetinaNet (developed by Facebook AI Research) had a considerably higher ADR and PDR than the group that had a colonoscopy without AI support [25]. The advantages of AI-assisted colonoscopy are presented in Figure 1. 

Endoscopic pictures can now be analyzed by AI-based algorithms, which have a high accuracy rate and minimal inter-observer variation in identifying neoplasms. EndoBRAIN (Cybernet Systems Co., Ltd., Tokyo, Japan)** **analyzes endocytoscopic observations. It inspects stained cell nuclei and the crypt structure to detect neoplastic cells. Kudo et al. conducted a multicenter study to assess the diagnostic accuracy of EndoBRAIN. This AI-based system identifies colon neoplasms by analyzing the microvessels, crypt structure, and cell nuclei in endoscopic images. EndoBRAIN efficiently differentiated between neoplastic and non-neoplastic lesions in endocytoscopic narrow-band images and stained endocytoscopic images, using pathology findings as the benchmark [26].

It has been demonstrated that both AI and distal attachment devices increase the rate of adenoma diagnosis and decrease the number of misses during colonoscopies. When compared with AI alone, Lui et al. discovered that Endocuff Vision™ plus AI further increases the detection rates of different colonic lesions [27].

Convolutional neural networks (CNNs) can analyze endoscopic pictures and identify polyps with high accuracy, reducing variability among endoscopists to increase optical diagnosis accuracy and making colonoscopy more efficient and cost-effective [28]. Another area where AI is expected to significantly impact regular clinical practice is lesion characterization and "optical diagnosis." To avoid pathology costs, two synergistic tactics can be used: "leave-in-situ" for <5 mm hyperplastic lesions in the rectosigmoid tract and "resect and discard" for smaller lesions [29].

Rondonotti et al. assessed the effectiveness of real-time AI-assisted optical diagnosis for correctly identifying small (≤5 mm) rectosigmoid polyps (DRSPs) during colonoscopy, supporting a "leave-in-situ" approach, where non-cancerous polyps are left without removal. The AI tool CAD EYE assisted endoscopists with varied skill levels in classifying DRSPs as adenomas or non-adenomas. AI-assisted optical diagnosis achieves the accuracy required for "leave-in-situ" techniques in DRSPs, consistent with guideline-based surveillance recommendations. While AI helps benefit non-expert endoscopists, it does not negate the necessity of clinical experience and decision-making confidence [30].

In comparison to optical colonoscopy (OC), both CCE and CTC show high sensitivity and specificity in detecting colonic polyps. CCE is more sensitive in detecting polyps smaller than a centimeter [31,32]. Introducing and enhancing algorithm-based capsule abortion localization may lead to higher rates of overall complete investigation following incomplete OC [31].

Despite being a useful procedure, CCE is underutilized because of the large number of pictures it generates, which makes analysis expensive and time-consuming. This could be resolved by advances in AI, which streamline image analysis and improve lesion detection accuracy, as demonstrated by real-time diagnostic technologies used in colonoscopies. AI will initially assist human validators in analyzing CCE photos. With increasing accuracy, AI can handle more minor instances independently and remotely deliver patient results. This strategy would improve patient outcomes by facilitating earlier intervention and making CRC screening more patient-centered, efficient, and accessible [33]. 

Autonomous AI outperformed AI-assisted human diagnosis regarding visual diagnosis accuracy; however, both methods were moderate. Autonomous AI showed greater alignment with recommended surveillance intervals, implying possible improvements in consistency and efficiency for surveillance planning [34]. 

Nevertheless, there are some difficulties and negative points around AI. The application of AI has been hindered by the heterogeneity of the AI models and study design, as well as a need for long-term outcomes. Furthermore, less experienced endoscopists can be passively influenced in their diagnoses and decisions by erroneous AI predictions because only a well-trained physician can accept or reject the interpretation provided by AI. Therefore, this could begin a new generation of technology-dependent endoscopists [8].

In addition to that, a study showed that even AI misses approximately 20% of adenomas and missed adenomas and polyps constitute 50% of the cancers that are identified within a short interval after a negative colonoscopy for cancer. These lesions that were not detected are not even seen on the screen, meaning that their locations are behind a difficult flexure position or hidden under fecal contents [10,27]. Another type of lesion that can pass undetected is a flat lesion. This is worrying because flat lesions (sessile serrated lesions and non-granular laterally spreading tumors) are associated with advanced histology. A patient's case report showed the CADe system, such as ENDO-AID software (Olympus Medical Systems Corp., Tokyo, Japan) in combination with the EVIS X1 video column (Olympus Medical Systems Corp., Tokyo, Japan), with difficulties detecting an adenocarcinoma invading the muscularis mucosae [35].

Despite that, the CADx system has been developed to predict polyp histology, as research in this field has been highly focused on in the last few years. The computational analysis interprets the content of the medical image by assigning a label to the image itself and then classifying the polyp into a category (e.g., hyperplastic, adenoma, etc.). These systems' sensitivity, specificity, feasibility, accuracy, positive predictive value, and negative predictive value have been studied [18].

A study used the WavSTAT4 (SpectraScience Inc., San Diego, California, USA) optical biopsy forceps system with laser-induced autofluorescence spectroscopy to anticipate the histology of diminutive polyps (≤5 mm). A total of 137 lesions were analyzed, and the accuracy result was 84.7%, with 81.8% sensitivity, 85.2% specificity, and 96.1% NPV. Another study used a narrow-band imaging (NBI) magnification colonoscopy system for a CAD real-time image recognition system to verify 118 colorectal lesions, and all the results (accuracy, sensitivity, specificity, positive predictive value, NPV) were above 93% [18].

A third study used ultra-magnifying colonoscopies associated with NBI or methylene blue staining modes, which allowed both visualization and microvascular and cellular imaging of polyps in the colon. Four hundred sixty-six diminutive polyps were analyzed, and the accuracy was 98.1%, with an NPV of 96.4% for methylene blue staining modes and 96.5% accuracy for NBI modes [18]. The technologies are rapidly evolving, and they promise to achieve valuable endoscopic innovations to characterize and classify small polyps. The technologies will also avoid the resection of non-hyperplastic/non-neoplastic polyps (that rarely involve malignant forms), resulting in lower financial expenditure, unnecessary exposure of healthy tissue to intervention, and saving time (related to the time that would be wasted in the observation and the resection of the lesions) [18].

As long as trained endoscopists present accuracy smaller than 80% to characterize and classify small polyps, there is the possibility to completely substitute the way to examine the pathological resected tissue with AI [18]. The key emerging is digital pathology for diagnosing and prognosis tumors [36]. Digital pathology is a cutting-edge technique that allows pathologists to see, examine, and distribute high-resolution whole-slide images (WSI) of tissue specimens in a digital format by digitizing conventional glass slides. Digital pathology promises to improve clinical practice by combining state-of-the-art software with imaging technology. For a more precise diagnosis, digital pathology enhances quality assurance and standardization and enables remote expert participation. AI in pathology greatly enhances cancer detection, classification, and prognosis through the automation of numerous processes. It furthers the translation of new biomarkers for therapeutic applications and improves the spatial analysis of the tumor microenvironment (TME) [37]. Accurate histologic diagnosis aspires because it would save time by not resecting lesions with low malignancy risk and prevent incomplete resection of lesions with high malignancy risk [38].

AI in diagnosis and preoperative assessment of colon cancer

Radiologists generally do clinical staging of CRC by evaluating contrast-enhanced CT images in colorectal cancer patients and MRI in rectal cancer patients [39]. Koçak et al. examine how radiomics and AI can be combined in medical imaging. Radiomics includes extracting a huge amount of quantitative information from medical images, whereas AI employs robust algorithms to analyze trends and make predictions based on the data [40]. The integration of these technologies improves data analysis for clinical decision-making [40,41]. Abbaspour et al. evaluated the utility of radiomics-based models for predicting lymph node metastasis (LNM) in patients with colorectal cancer. They concluded that radiomics has excellent potential to improve preoperative staging and management of colorectal cancer [41]. Bedrikovetski et al. emphasize that AI has promising applications for lymph node staging but highlight the necessity for more robust multicenter trials and standardized clinical implementation procedures [42].

Chen et al. investigate how dual-energy CT (DECT) can assess lymph node metastases in various cancer types. By employing two energy levels to increase material distinction, DECT offers more image detail than traditional CT. According to the review, it can identify small or micrometastatic lymph nodes, which are hard to find with traditional CT. It has been demonstrated that combining these factors with radiomics or machine learning techniques can improve the prediction and diagnosis of tumors such as thyroid and colorectal cancer [43]. The use of AI to address issues with colorectal cancer liver metastases (CRLM) is examined by Rompianesi et al. It demonstrates how AI tools like ML and radiomics can improve treatment plans, save expenses, and increase diagnostic accuracy [44].

Wang et al. assess the performance of ML-enhanced radiomics models in predicting microsatellite instability (MSI) in colorectal cancer. The key findings show that the models have great potential, with area under the receiver operating characteristic curve (AUROC) values ranging from 0.75 to 0.95, particularly when combining radiomics and clinical data. The study emphasizes the clinical value of these techniques in customizing therapy for MSI-high individuals [45]. He et al. emphasize how non-invasive, preoperative diagnostics that combine radionics and machine learning can help with better staging and individualized treatment planning for colorectal cancer [46].

Quero et al. investigate how AI can be included in CRC surgery. It emphasizes how AI is used in surgical decision-making - by evaluating imaging data, AI helps with preoperative planning by identifying tumor characteristics, determining surgical outcomes, and estimating risks [47]. Several applications, including image analysis, screening, staging, and prognosis prediction, are discussed, along with how AI technologies, particularly ML and DL, help improve the precision and effectiveness of CRC detection. AI has also shown incredible potential in the early identification of colorectal cancer, particularly when paired with sophisticated imaging methods like endoscopy, radiography, and histopathology. It also makes more accurate tumor staging and treatment response prediction possible, eventually advancing personalized medicine [48,49].

Robotic-assisted vs. conventional laparoscopic surgery in intraoperative colon cancer management: efficiency, safety and outcomes

There are three main approaches for colorectal cancer surgery these are open, laparoscopic, and robotic. Laparoscopic surgery for colorectal cancer offers many advantages over open surgery, like smaller incisions, fewer postoperative infections, and quicker recovery. However, despite many advantages over open surgery, it has some limitations like 2D view, limited dexterity, and a need for assistant-dependent camera handling along with the time needed to achieve proficiency by surgeons to perform the surgery efficiently. On the contrary, robotic-assisted surgery and laparoscopic surgery have many similar advantages along with many differences. In recent times, robotic-assisted surgery has rapidly emerged as a revolutionary approach in the surgical management of colorectal cancer, offering distinct advantages over conventional laparoscopic surgery (CLS) and gaining popularity, particularly in complex procedures like intracorporeal anastomosis. Regarding the mechanism of the procedure, laparoscopic is done using a scope by hand using a 2D camera near the patient body.

In contrast, robotic surgeons operate robot arms using instruments and a 3D camera outside the operation room, and conventional is open surgery. In recent years, robotic and laparoscopic surgeries have gained more popularity due to various advantages over open and laparoscopic surgery. The robotic system's high-definition 3D and flexibility offer enhanced precision that enables surgeons to perform intricate tasks with greater accuracy, especially in complex regions like the right colon and these features are particularly valuable when performing complex procedures, such as intracorporeal anastomosis (IA), in which precision is critical for reducing complications like leakage or stricture formation. Weber et al. first described a robot-assisted colon surgery (RCS) report [49], and subsequent research highlighted the effectiveness of this technique [50].** **RCS was introduced to improve dissection by viewing the pelvis in 3D [51-54], successfully simplifying the dissection for surgeons. Robotic surgery was approved for general surgery applications in July 2020 by the US Food and Drug Administration (FDA), representing that advanced technology has been integrated to enhance surgical procedures further and reduce associated overall complications. There have been various technological developments in robotic surgery, such as tremor reduction and enhanced camera guidance, which have made it more refined, proving it to be a promising alternative that enhances accuracy with advanced maneuverability and other patient benefits, such as reduced complications [55-57]. Robotic-assisted surgery has demonstrated its benefits for procedures like hemicolectomy for ascending colon cancer in patients with anatomic anomalies such as situs inversus totalis, as highlighted in the study. Situs inversus totalis (SIT) is a rare congenital anomaly characterized by reversed thoracic and abdominal organ positioning that makes the laparoscopic surgical approach difficult for colorectal cancer removal. The case study used robot-assisted right hemicolectomy for ascending colon cancer in a 74-year-old female with SIT, and it reported robotic-assisted surgery to be a safe and effective surgical approach due to some advantages over laparoscopic like multi-joint forceps and dominant hand usage that improves precision, port placement modification by using mirror-image trocar positioning that ensures a standard surgical view and using a retraction arm with the non-dominant hand was feasible that enabled surgeons to efficiently manage complex anatomic anomalies for colorectal surgery [58].

Laparoscopy was introduced in the 1990s, and it is preferred for 40-50% of all colorectal surgery routes for benign or malignant colon malignancy [59-64]. Laparoscopy colorectal surgery (LCS) is associated with low morbidity, short hospital stay, reduced analgesic use, and quick recovery of bowel function versus open surgery [65-67]. However, the big question remains if robotic-assisted surgery is better than laparoscopic-assisted surgery, and the answer to this is vague as it has not been examined in randomized controlled trials except for Park et al. [25] that showed no significant difference in peri or postoperative outcomes between two assisted surgeries for colon cancer management. Differences like high cost and operation time make laparoscopic more favorable for most of the population [25].

We need more data to decide which is the best approach. We have data from a mixed-stage population for colorectal surgery [66-68], and systemic review and meta-analysis have not shown any significant difference in recovery. Besides low morbidity rates in right-sided colectomies performed by RCS versus LCS in a heterogeneous population, to our knowledge, no meta-analyses have been conducted to determine the overall effects of RCS in colonic cancer surgery [69-70]. To gain more information on short-term outcomes of RCS versus LCS for colon cancer, such as operative time and length of stay was studied in the study using Preferred Reporting Items for Systematic Reviews and Meta-Analyses (PRISMA) guidelines and Meta-analysis of Observational Studies in Epidemiology (MOOSE) checklist [71,72]. This study reported primary like anastomotic leakage rate, conversion to open surgery, operative time, and length of hospital stay.

In contrast, secondary outcomes assessed overall complication rates, including factors like time to first flatus, oral diet resume time, intraoperative blood loss or postoperative bleeding, surgical site infections or abscess formation, bowel obstruction, lymph node harvest, 30-day mortality, Clavien-Dindo classification, and both surgical and medical complications [73], was assessed for bias or quality of evidence Grading of Recommendations Assessment, Development, and Evaluation (GRADE) criteria by three reviewers and found to have low risk of bias for randomization, data outcome and reporting. It included 13,799 patients, with 1,862 (13%) undergoing robot-assisted and 12,231 (87%) laparoscopic colon surgery. Many studies were observational [74-96]. 

For meta-analysis, 20 studies were taken that covered 13,799 patients, 1,740 underwent RCS, and 12,059 underwent LCS. It concluded that there were significant differences in preoperative characteristics between the groups, specifically relating to prior abdominal surgery and male gender. However, there was no statistically significant difference in age, BMI, tumor stage (T-stage ≥3), or American Society of Anesthesiologists Physical Status Classification System (ASA) score ≥3. Overall, the study results showed that RCS is superior to LCS in certain aspects, such as that anastomosis leaks were low in RCS, and so were overall complications. Additionally, postoperative factors like regular diet intake were much shorter in patients undergoing RCS, and most notable of all was its benefit in right-sided colonic cancer treatment [25]. Despite all the promising results, there is still a limited presence of prospective studies and randomized control trials that impact the strength of evidence to be widely adopted. To conclude, RCS is still a "work in progress" with the possibility of widespread use in the near future.

Another research was conducted to understand more in-depth and find the answer to the question of comparison between the two; that also looked into all the primary and secondary factors, and it concluded that even though robotic surgery has some advantages in terms of length of recovery and lymph node harvest and conversion rate to open surgery in which robotic surgery takes high precedence but overall efficacy and safety showed no major difference. The research showed that both are equally related to better outcomes for patients compared to open surgery, with one exception where laparoscopic excels over robotic op: operation time that significantly increased during robotic surgery, which may be due to factors such as surgeon experience and ease of use with technology use that needs more intense training. Ultimately, it is concluded that either type of surgery is equally effective and that the procedure selection depends on patient factors, surgeon expertise, and affordability.

AI in postoperative care

Robotic-assisted surgery achieves comparable oncological outcomes, such as lymph node yield, margin clearance, and surgical safety (30-day mortality), to CLS in colorectal cancer resections. Additionally, it offers statistically significant advantages over CLS regarding conversion rates, wound infections, all-cause mortality, and shorter hospital stays. The technological advancements of robotic-assisted surgery allow better access to difficult-to-reach anatomical areas, resulting in a markedly lower conversion rate (0% in robotic-assisted surgery vs. 16.4% in CLS). However, a significant drawback is its high financial cost, which can be mitigated by its association with shorter hospital stays (approximately two days), fewer complications, and favorable oncological results [97].

Compared to laparoscopic surgery, postoperative outcomes are generally better with robotic surgery, as evidenced by a lower incidence of anastomotic leaks (approximately 4%) and shorter recovery times. However, these outcomes are influenced by factors such as tumor location, the surgeon's expertise, and patient comorbidities. In right colectomy (RC), the robotic approach significantly reduced postoperative ileus rates compared to laparoscopy (9.0% vs. 11.6%; p<0.001), while in left colectomy (LC), the difference was not statistically significant (7.9% vs. 8.6%; p=0.170). The rates of anastomotic leaks, overall complications, significant morbidity, and 30-day mortality were similar between robotic and laparoscopic approaches for RC and LC. However, robotic RC and LC achieved higher textbook outcome rates than laparoscopy (71.0% vs. 64.0% for RC and 74.6% vs. 68.1% for LC; p<0.001), mainly due to shorter hospital stays and lower complication rates. In contrast, for lower anterior resection (LAR), robotic surgery showed higher rates of anastomotic leaks (3.4% vs. 2.4%), postoperative ileus (11.9% vs. 10.5%), and significant morbidity (7.1% vs. 5.8%) compared to laparoscopy. Despite this, the two approaches had comparable overall complication rates, textbook outcome rates (68% vs. 67%), and 30-day mortality (0.4% vs. 0.4%) [98]. These results included cases of low, middle, and high rectal cancers. According to Feng et al., robotic surgery for lower anterior resection (LAR) in middle and low rectal cancer demonstrated superior oncological quality of resection compared to conventional laparoscopic surgery. It was also associated with reduced surgical trauma and improved postoperative recovery [96]. Trastulli et al. conducted a meta-analysis which included 4,148 patients. All these patients had either undergone robotic or laparoscopic colectomy. The study implicated the advantages of robotic colectomy over laparoscopic. Although it is expensive, it caused lower blood loss, faster recovery, and less hospital stay due to a lower risk of complications [99].

The outcomes of robotic surgery can also be evaluated by assessing the performance of robotic colorectal cancer units using Textbook Oncological Outcome (TOO). This metric combines all "ideal" postoperative clinical and oncological results from patient and physician perspectives. In a multicenter retrospective study involving 501 patients who underwent robotic colorectal cancer surgery, 388 patients (77.4%) achieved TOO. TOO was defined as the absence of conversion to open surgery, no Clavien-Dindo (CD) complications ≥ grade 3, a length of stay (LOS) ≤14 days, no 30-day readmission, no 30-day mortality, and R0 resection. The primary reasons for not meeting to achieve TOO were CD ≥3 postoperative complications, reported in 46 patients (9.2%), and a prolonged LOS exceeding 14 days, observed in 55 patients (11%) [100].

Postoperative systemic inflammatory response in RCS and LCS 

Both robotic and laparoscopic surgery have shaped colorectal surgeries in different directions compared to conventional open surgery, marking the beginning of the modern era of surgical approach for CRC management. Despite many positive outcomes studied in various types of research, both have their challenges, as laparoscopic surgery is associated with a prolonged learning curve, suboptimal ergonomics, tremors, and camera position issues. In contrast, robotic surgery requires higher operating time and high technological knowledge. Along with all functional challenges, we wanted to gain more understanding of infections related to each surgery. The biggest challenge in patient postoperative care is infection-related complications, and it is vital to understand the factors that increase the infection risk in postoperative care, including length of hospital stay and bowel recovery time, two of the important considerations in deciding the outcome. To our knowledge, no RCT studies have been conducted that compare systemic or peritoneal inflammatory responses between two surgeries in colon cancer management. 

Post-surgery, one of the critical factors in surgical outcome is the inflammatory response, which decides a patient's recovery. A systemic inflammatory response is a widespread and life-threatening response to various factors like infection, trauma, burns, or pancreatitis. The pathophysiology of systemic inflammatory response syndrome (SIRS) includes the widespread response by release of pro-inflammatory cytokines such as interleukin 1 beta (IL-1β), interleukin 6 (IL-6), and tumor necrosis factor (TNF), which play a vital role in wound healing with IL-6-associated worse outcomes that can lead to the development of SIRS that has higher mortality rate. There are guidelines developed to recognize SIRS; the criteria are as follows: temperature (fever >38°C or hypothermia <36°C), heart rate (>90 bpm), respiratory rate (>20 bpm or partial pressure of carbon dioxide, PaCO_2_ of <32 mmHg). SIRS has a high index of fatality and can rapidly progress to sepsis, organ failure, or shock if not managed timely. There is not enough data due to a lack of RCT studies. However, some non-randomized clinical trials suggest robot-assisted colorectal surgery has an advantage due to faster bowel function recovery and less hospital stay duration, along with factors like enhanced tissue exposure in 3D-vision, wristed instruments, and tremor reduction that has minimized the inflammatory stress response in robotic-assisted surgery for colon cancer compared to laparoscopic surgery [101-104].

Surgical stress in robotic-assisted versus laparoscopic surgery is still an ongoing research topic, and more evidence is needed to decide whether one technique is superior to the other. Many studies have been conducted to compare robotic versus open surgery, but more information is needed to compare two minimally invasive techniques [105-109]. 

Only one randomized control trial study was conducted that evaluated the systemic and peritoneal inflammatory stress responses induced by minimally invasive surgery in colon cancer resections. A comparison was made between the two techniques. The study was conducted at the Surgical Department, Hospital Sønderjylland, University Hospital of Southern Denmark, that required elective robot-assisted or laparoscopic surgery for right-sided, left-sided, or sigmoid colon cancer [100]. It also included specific interventions in surgical procedures like prophylactic antibiotics post-surgery, no bowel preparation before surgery, and the da Vinci system for robotic surgery, with all the surgeries performed by highly trained surgeons in both techniques for required hours and cases [110-113]. Apart from surgical interventions, the study also focused on peritoneal fluid stats by inserting 61 high-cut-off microdialysis catheters intraoperatively at the end of surgery that collected peritoneal fluid for further analysis to gain a more in-depth understanding of SIRS in minimally invasive surgeries. At the end of the study, many endpoints like genetic, environmental, systemic vitals, interleukin functions, and postoperative health were analyzed.

The study collected data to analyze inflammatory mediators like CRP. It showed that in 298 patients who underwent robotic-assisted or laparoscopic colorectal surgery, CRP level was 19% higher in laparoscopic compared to robotic surgery with both interpersonal and intrapersonal variation of 0.63 and 0.34, respectively [100]. Many studies concluded that robotic surgery has less immune stress than open surgery, but we do not have enough reliable data compared to laparoscopic surgery [114]. It was found that the CRP level in robotic surgery is significantly lower due to minimal tissue traumatization compared to laparoscopic surgery. CRP plays an important role in inflammation, and surgeries like open and laparoscopic both have tissue traumatization (to some extent in laparoscopic vs. robotic) that causes increased activation of macrophages intraperitoneally, stimulating an increase in cytokine levels of IL-1, IL-6, and tumor necrosis factor (TNF-α) [114]. However, many of these studies are observational and have high chances of selection bias, which further affects the data collection and interpretation. For more accurate data, we need more randomized control trials to learn more and to conclude which should be the surgery of choice for CRC - robotic or laparoscopic surgery. 

Challenges and limitations

The potential benefits of robotic-assisted surgery continue to be highlighted as more research shows benefits such as reduced blood loss and improved postoperative recovery. However, several challenges and roadblocks still need to be addressed, preventing its widespread adoption.

The adoption of robotic surgery remains limited in many countries, highlighting numerous existing barriers. For instance, the Netherlands reported that only 6.9% of surgeries related to colon cancer were robotic-assisted [115], while in England, the number of robotic surgeries for colon cancer increased from 0.1% to only 2.7% between 2007 and 2021. One of the primary reasons for the slow adoption is the shortage of trained surgeons proficient in robotic surgery [116]. This is exacerbated by a steep learning curve, which may initially result in longer procedure times and higher complication rates. Unfortunately, many countries lack systemized training programs that could help overcome this initial barrier. Efficient training requires surgeons to have regular access to robotic equipment, ensuring adequate familiarity to enhance their skills [117]. Additionally, a dedicated surgical team, including trained assistants and technicians, must be developed, further complicating the training process.

Beyond the technical learning curve, financial constraints pose a major obstacle to implementation. Institutions face significant upfront costs for acquiring robotic surgical systems, which can range between $1 million and $2.5 million per unit, depending on the manufacturer and specific features. Additionally, there are substantial recurring costs, including maintenance, software updates, and instrument replacement, which can exceed $100,000 annually per system. A study comparing five different surgical approaches found that robotic colorectal surgery resulted in the highest total and surgical costs [118]. Furthermore, per-procedure costs for robotic surgery can be higher due to disposable instrument expenses, which are estimated at $2,000 to $3,500 per case. As such, the financial burden of robotic surgery may deter institutions from investing in the necessary equipment and training programs. Hospitals may also require stronger evidence on the cost-effectiveness of robotic surgery before committing to large-scale implementation [116].

Another major limitation is the insufficient clinical evidence supporting the superiority of robotic-assisted surgery over conventional techniques, particularly in terms of long-term outcomes. While robotic-assisted procedures have demonstrated benefits such as reduced intraoperative blood loss and shorter hospital stays, there remains a lack of large-scale, randomized clinical trials proving a definitive survival advantage over other treatment modalities for colon cancer. Some data suggest that survival outcomes for colon cancer metastasis may be less favorable, further complicating the justification for broader adoption [119]. Consequently, hospitals may hesitate to invest in robotic surgery without compelling evidence of improved patient survival and overall treatment efficacy.

In addition to financial and clinical concerns, regulatory challenges hinder the widespread adoption of robotic-assisted surgery. Regulatory bodies in different countries impose varying requirements for approving robotic surgical systems, which can delay market entry and increase costs. For example, in the United States, robotic surgical platforms must undergo rigorous FDA approval processes, requiring extensive clinical trial data before they can be commercially available. Similarly, in Europe, the CE marking process necessitates compliance with the Medical Device Regulation (MDR), which can be time-consuming and resource-intensive. These regulatory hurdles can slow technological advancements and limit accessibility in certain regions.

Systematic biases and structural barriers further limit equitable access to robotic surgery, reducing its potential benefits for the broader population. Disparities in access are influenced by factors such as insurance coverage, geographic location, and socioeconomic status. Notably, racial and ethnic minorities are disproportionately affected, with one study reporting that Black and American Indian, Aleutian, and Eskimo (AIAE) patients were less likely to undergo robotic surgery compared to White patients [120]. Given the potential for robotic surgery to improve clinical outcomes in colon cancer, restricted access could exacerbate survival disparities, raising significant ethical and public health concerns [121].

Addressing these barriers requires a multi-faceted approach, including standardized training programs, financial incentives for hospitals, stronger clinical evidence, and regulatory reforms to streamline approvals. Until these issues are adequately addressed, the widespread adoption of robotic-assisted surgery will remain a challenge despite its potential advantages.

Future directions

Having confirmation from these studies substantiates to institutions that it is worth investing in robotic surgery, allowing more people to benefit from it. Future efforts should be directed at randomized controlled trials on comparative benefits and the wider role of AI, postoperative monitoring, and individualized treatment, among others. However, this will not come without its challenges, such as the costs involved and the availability of specialized training. Even then, AI technology must be part of, rather than a substitute for, the clinical expertise in ensuring optimum and equitable care, especially for underprivileged countries where access to this sort of technology is limited [112-115].

Some countries, like the Netherlands, are already trying to standardize robotic surgery, which could drive broader adoption and ensure consistent care across regions [116]. However, international collaboration between medical centers is needed to ensure a global standard is established [121]. This will allow for universally accepted guidelines that can be applied across diverse healthcare settings.

One key challenge that remains is addressing the disparity in outcomes among different demographic groups, particularly Black patients, who have been found to experience worse surgical outcomes. There is, therefore, a need for a change in the healthcare system at a systemic level so that these disparities can be addressed. This is so it can be ensured that all patients, regardless of background, can access and benefit from robotic surgery [120]. 

Another important consideration for the future of robotic surgery is the experience level of the surgical facility. Rather than individual surgeon expertise, institutional expertise significantly reduces operative times and improves surgical outcomes [119]. One primary concern for implementing AI is data privacy and security. Safeguarding patients' information and avoiding breaches of confidentiality are of utmost importance. The data used to train the AI algorithm has to be screened to detect potential bias since interpreting the results as per specific patient populations could result in discriminatory results. Patient must be fully informed about how AI tools can be integrated into their care. While AI can enhance diagnostic accuracy, healthcare providers may become too dependent, potentially undermining their clinical judgment and intuition. It is essential to strike a balance between AI tools and the expertise of clinicians [122].

With the evolution of robotic surgery, the development of specialized training programs for surgical teams and healthcare facilities will be essential to improving the quality and consistency of care. In conclusion, while robotic surgery for colon cancer holds great promise, further research, standardization, and system-level transformations are needed to ensure widespread adoption.

## Conclusions

Artificial intelligence in colonoscopy has demonstrated potential in improving polyp detection, lowering miss rates, and increasing adenoma detection rates, particularly for flat or smaller polyps. Deep learning algorithms and other AI-powered tools can assist endoscopists by decreasing mistakes and variability, which results in more precise diagnostics and less overlooked neoplasia. AI can also help with optical diagnosis, assisting in the decision-making process regarding the safe in situ placement of non-cancerous lesions, hence minimizing the need for needless polypectomies. Future efforts should be directed at randomized controlled trials on comparative benefits and the broader role of artificial intelligence, postoperative monitoring, and individualized treatment. However, this will not come without challenges, such as the costs involved and the availability of specialized training. Even then, AI technology must be part of, rather than a substitute for, the clinical expertise in ensuring optimum and equitable care, especially for underprivileged countries with limited access to this technology.
